# Real-world risk assessment and treatment initiation among patients with myelofibrosis at community oncology practices in the United States

**DOI:** 10.1007/s00277-020-04055-w

**Published:** 2020-05-07

**Authors:** Srdan Verstovsek, Jingbo Yu, Jonathan K. Kish, Dilan Paranagama, Jill Kaufman, Callan Myerscough, Michael R. Grunwald, Philomena Colucci, Ruben Mesa

**Affiliations:** 1 Department of Leukemia, The University of Texas MD Anderson Cancer Center, 1515 Holcombe Blvd, Houston, TX 77030 USA; 2 Incyte Corporation, Wilmington, DE USA; 3 Cardinal Health Specialty Solutions, Dublin, OH USA; 4 Present Address: Ohio State University, Columbus, OH USA; 5 Levine Cancer Institute, Atrium Health, Charlotte, NC USA; 6 UT Health San Antonio Cancer Center, San Antonio, TX USA

**Keywords:** Myelofibrosis, Risk stratification, IPSS, Chart review, Treatment patterns

## Abstract

Myelofibrosis (MF) is a chronic myeloproliferative neoplasm with a prevalence of 4 to 6 per 100,000 people in the USA. Treatment recommendations are risk-adapted. This study was conducted to evaluate how physicians risk-stratify patients at the time of MF diagnosis, the accuracy of the risk stratification, and its effect on treatment selection. Medical charts were reviewed at US community hematology/oncology practices in the Cardinal Health Oncology Provider Extended Network; patient clinical characteristics, risk stratification, and treatment data were collected. Physician-assigned risk categorizations were compared with data-derived risk categorizations based on the International Prognostic Scoring System, the system recommended at diagnosis. A total of 491 patients diagnosed with MF between 2012 and 2016 (mean [SD] age at diagnosis, 65.4 [11.8] years; 54.8% male, 69.2% with primary MF) were included. Risk categorization was not assigned for 30.1% of patients. Of the patients with a physician-assigned risk categorization (*n* = 343), a scoring system was used in 49.9%. Compared with data-derived risk categorizations, 42.9% of physician-assigned risk categorizations were incorrect; 85.0% of incorrect physician-assigned risk categorizations were underestimations. Notably, 38.5% of patients with data-derived intermediate- or high-risk categorizations did not initiate treatment within 120 days of diagnosis. Among patients with data-derived intermediate risk, those with an underestimated physician-assigned risk categorization were significantly less likely to receive treatment within 120 days of diagnosis (51.6% with correct physician-assigned categorization vs 18.5% with underestimated risk categorization; *P* = 0.0023). These results highlight the gap in risk assessment and the importance of accurate risk stratification at diagnosis.

**Electronic supplementary material:**

The online version of this article (10.1007/s00277-020-04055-w) contains supplementary material, which is available to authorized users.

## Background

Myelofibrosis (MF) is a myeloproliferative neoplasm characterized by bone marrow fibrosis, extramedullary hematopoiesis, leukoerythroblastosis, and, frequently, the presence of *JAK2*, *CALR*, or *MPL* driver mutations [1, 2]. Clinical manifestations often include anemia, splenomegaly, and constitutional symptoms (e.g., weight loss, fever, night sweats) [3, 4]. In the USA, the estimated MF prevalence is between 4 and 6 per 100,000 people [5], and the median survival in patients with primary MF ranges from 2 to 11 years [3]. This wide range in survival time is indicative of the heterogeneity of disease severity and patient prognosis, which may be estimated with a number of scoring systems. The National Comprehensive Cancer Network (NCCN) Clinical Practice Guidelines in Oncology (NCCN Guidelines^®^) for Myeloproliferative Neoplasms, which became available in 2017, recommend the use of the International Prognostic Scoring System (IPSS) for risk stratification at diagnosis (Table 1). IPSS risk stratification assigns 1 point for each of the following prognostic variables: age > 65 years, white blood cell (WBC) count > 25 × 10^9^/L, hemoglobin < 10 g/dL, peripheral blood blasts ≥ 1%, and presence of constitutional symptoms. However, stratification models are evolving. In addition to the IPSS, the Dynamic IPSS (DIPSS) and the DIPSS-Plus, also referenced in the NCCN Guidelines MF algorithms, are recommended for use during treatment rather than at diagnosis [6, 7]. Mutation-enhanced IPSS (MIPSS70) can be used for risk stratification of patients with primary MF aged ≤ 70 years [8], and the Myelofibrosis Secondary to Polycythemia Vera and Essential Thrombocythemia-Prognostic Model (MYSEC-PM) has been developed for risk stratification of patients with secondary MF [9].

**Table 1:** Risk categorization per International Prognostic Scoring System (IPSS) for patients with myelofibrosis (MF) [3]

	Points	
	0	1
Prognostic variable		
Age, years	≤ 65	> 65
WBC count, × 10^9^/L	≤ 25	> 25
Hemoglobin, g/dL	≥ 10	< 10
Peripheral blood blast, %	< 1	≥ 1
Constitutional symptoms	No	Yes
	Points	
Risk group		
Low	0	
Intermediate 1	1	
Intermediate 2	2	
High	≥ 3	

*WBC* white blood cell

Treatment recommendations in the NCCN Guidelines are risk-adapted, reinforcing the importance of accurately assessing patient risk categorization at diagnosis [2]. Despite the central role that accurate risk categorization plays at the time of diagnosis with respect to treatment selection and prognostication, limited data are available on how initial risk is assessed in real-world clinical practice [10]. This retrospective medical chart review evaluated how patients with MF were risk-stratified at diagnosis, the impact that the use of a prognostic risk scoring system had on the accuracy of risk stratification, and the effect of the accuracy of risk stratification on the timing of treatment initiation at community hematology/oncology practices in the USA.

## Methods

### Study design and patients

This retrospective medical chart review was conducted at US community hematology/oncology practices in the Cardinal Health Oncology Provider Extended Network (OPEN), which includes practices across the country. Adult patients diagnosed with primary MF, post-polycythemia vera (PV) MF, or post-essential thrombocythemia (ET) MF between January 2012 and December 2016 and who received care for ≥ 6 months (or died within 6 months of diagnosis) at the participating practices were included. Between May and July of 2018, physicians extracted data documented at the time of MF diagnosis and during follow-up visits from medical charts for patients under their care and entered the data into an electronic case report form (eCRF). The eCRF included questions pertaining to clinical characteristics, risk stratification method used and the risk level assigned (if applicable) by treating physicians at the time of diagnosis, and treatments administered for MF. Clinical characteristics included patient demographics, MF type, mutation testing, spleen status, transfusion dependence, MF-related symptoms, hemoglobin level, WBC count, platelet count, peripheral blood blast percentage, and medical history. Risk stratification method choices included IPSS, DIPSS, DIPSS-Plus, and clinical/qualitative judgment (i.e., the provider indicated that a formal prognostic scoring system was not used). When a specific risk stratification method was used, data were collected regarding the risk category assigned and the risk assessment score, if documented. For patients whose risk was assigned based on clinical/qualitative judgment, the risk category choices were low, intermediate, or high risk; the eCRF did not distinguish between intermediate 1 and intermediate 2. Physician-assessed risk categorization used for this analysis was the score or risk categorization documented in the medical record within 30 days of the diagnosis of MF.

A data-derived IPSS risk categorization was determined for all patients with individual risk variables that included age, WBC count, hemoglobin level, peripheral blast count, and constitutional symptoms (e.g., weight loss, fever, night sweats) at the time of diagnosis. To assess the accuracy of the physician-assigned risk categorization at diagnosis, this data-derived IPSS risk categorization was compared with the physician-assigned risk categorization. In addition, a data-derived risk categorization was determined with risk factor data collected in eCRFs with the same scoring tool (i.e., IPSS, DIPSS, or DIPSS-Plus) used by the treating physician at the time of diagnosis. For providers who indicated that they used their “clinical/qualitative judgment” as opposed to a specific prognostic scoring system when assigning a risk category, the data-derived risk categorizations were limited to low, intermediate, or high risk as calculated with the IPSS.

In the analysis, patients were defined as having initiated treatment upon diagnosis if any MF-directed treatment (hydroxyurea (HU), interferon (IFN) alfa-2b, pegylated-IFN alfa-2b, ruxolitinib, a clinical trial with an investigational drug, or referral for hematopoietic cell transplantation (HCT)) was initiated within 120 days of the date of MF diagnosis. Fedratinib was approved for the treatment of MF after the inclusion period of this study and, consequently, was not included as MF-directed therapy but was captured as an investigational drug in a clinical trial [11]. To describe the association of accurate risk categorization and treatment initiation at diagnosis (as defined above), the proportion of patients with a correctly assigned risk categorization who initiated treatment within 120 days was compared with the proportion of patients whose risk was incorrectly assigned versus patients whose risk was underestimated based on the data-derived IPSS score.

### Statistics

Categorical measures were reported as frequency and percentage; continuous measures were described with mean and median. The discordance between physician-assigned risk categorization (low, intermediate, or high) and data-derived risk categorization was assessed with the Cohen’s kappa coefficient and 95% CI, and *P* value for the test of symmetry was used to assess the level of agreement between the two methods. The differences in the proportion of patients treated versus not treated at diagnosis by correct versus underestimated risk category was assessed by chi-square tests.

## Results

### Patients

Records from 491 patients with MF from 45 community hematology/oncology providers in OPEN were included (Table 2). Patients were predominantly white (65.2%) and male (54.8%), with a mean (SD) age at diagnosis of 65.4 (11.8) years. More patients had primary MF (69.2%) than post-PV (17.7%) or post-ET (13.0%) MF. Median (interquartile range) disease duration at the time of the study was 27.3 (19.1–40.2) months.

**Table 2:** Patient demographics and clinical characteristics at diagnosis as reported by physicians

	Patients, *N* = 491
Mean (SD) age at diagnosis, years	65.4 (11.8)
Sex, *n* (%)	
Male	269 (54.8)
Race, *n* (%)	
White	320 (65.2)
Black/African American	104 (21.2)
Asian	50 (10.2)
American Indian or Alaska Native	6 (1.2)
Native Hawaiian/Pacific Islander	5 (1.0)
Other	6 (1.2)
Geographic region of treating physician practice, *n* (%)	
South	16 (35.6)
West	12 (26.7)
Northeast	9 (20.0)
Midwest	8 (17.8)
Type of MF, *n* (%)	
Primary MF	340 (69.2)
Post-PV MF	87 (17.7)
Post-ET MF	64 (13.0)
Median (IQR) disease duration at the time of the study, months	27.3 (19.1–40.2)
Palpable spleen, *n* (%)	
Yes	374 (76.2)
No	116 (23.6)
Unknown	1 (0.2)
Spleen length, *n* (%)	
< 5 cm, spleen not palpable or barely palpable	76 (15.5)
≥ 5 cm but < 10 cm; spleen palpable below the coastal margin	152 (31.0)
≥ 10 to < 20 cm; spleen palpable between the coastal margin and the umbilicus	113 (23.0)
≥ 20 cm; spleen palpable near to the umbilicus or severe splenomegaly	32 (6.5)
Unknown	1 (0.2)
Transfusion dependent, *n* (%)	
Yes	124 (25.3)
No	366 (74.5)
Unknown	1 (0.2)
Symptomatic disease at diagnosis, *n* (%)	312 (63.5)
Lab values, *n* (%)	
Blood blast > 1%	299 (61.2)
Hemoglobin < 10 g/dL	291 (59.3)
WBC count > 25 × 10^9^/L	84 (17.1)
Platelets < 100 × 10^9^/L	161 (32.8)

*ET* essential thrombocythemia, *IQR* interquartile range, *MF* myelofibrosis, *PV* polycythemia vera, *WBC* white blood cell

### Physician-reported risk stratification at diagnosis

A risk category (low, intermediate, or high) was assigned by the physician at the time of MF diagnosis for 343 patients (69.9%; Table 3); a considerable proportion of patients (148/491 [30.1%]) did not have a risk category documented at the time of diagnosis. Of those patients assigned a risk category, approximately half received a risk assignment with a scoring system (171/343 [49.9%]). The most commonly used scoring system was DIPSS (83/171 [48.5%]), followed by IPSS (54/171 [31.6%]) and DIPSS-Plus (34/171 [19.9%]). The remainder of patients’ risk categorizations (172/343 [50.1%]) were assigned using clinical/qualitative judgment. Of the patients assigned a risk category by the physician at diagnosis, 42/343 (12.2%) were classified as low risk, 200/343 (58.3%) intermediate risk, and 101/343 (29.5%) high risk.

**Table 3:** Methods used and risk categories assigned by physicians at the time of myelofibrosis (MF) diagnosis

	Patients, *n* = 343
Method used for physician-assigned risk level	
IPSS	54 (15.7)
DIPSS	83 (24.2)
DIPSS-Plus	34 (9.9)
Clinical/qualitative judgment	172 (50.1)
Physician-assigned risk level	
Low	42 (12.2)
Intermediate	200 (58.3)
High	101 (29.5)

*DIPSS* Dynamic International Prognostic Scoring System, *IPSS* International Prognostic Scoring System

### Data-derived IPSS risk categorization

Among all 491 patients, the data-derived IPSS risk category was low for 32 patients (6.5%), intermediate for 207 (42.2%), and high for 250 (50.9%). For the 343 patients who received a physician-assigned risk category at diagnosis, 20 (5.8%) patients were categorized as low risk, 135 (39.3%) as intermediate risk, and 188 (54.8%) as high risk according to data-derived IPSS risk categorization (Table 4). Of the 148 patients who did not receive physician-assigned risk categorization at diagnosis, the data-derived IPSS risk category was low in 12 (8.1%) patients, intermediate in 72 (49.3%), and high in 62 (42.5%); risk category was incalculable in 2 (1.4%) patients owing to the absence of peripheral blast percentage at the time of diagnosis.

**Table 4:** Risk categorization at the time of myelofibrosis (MF) diagnosis, physician-assigned versus data-derived

	Total	Data-derived risk categorizations (IPSS only)*		
		Low	Intermediate	High
Physician-assigned risk category, *n* (row %)	343	20 (5.8)	135 (39.3)	188 (54.8)
Low	42	10 (23.8)	*26 (61.9)*	*6 (14.3)*
Intermediate	200	10 (5.0)	**97 (48.5)**	*93 (46.5)*
High	101	0	***12 (11.9)***	**89 (88.1)**
Incorrect risk categorization by physician, *n* (column %)	147 (42.9)	10 (50.0)	38 (28.1)	99 (52.7)
Underestimated, *n* (%)^†^	*125 (85.0)*	*–*	*26 (68.4)*	*99 (100.0)*
Overestimated, *n* (%)^†^	***22 (15.0)***	10 (100.0)	***12 (31.6)***	***–***
Risk not assigned by physician, *n* (row %)	148^‡^	12 (8.1)	72 (49.3)	62 (42.5)

*IPSS* International Prognostic Scoring System

*Cohen’s kappa (95% CI) = 0.2881 (0.2097–0.3664); *P* < 0.001

^†^Of incorrect total in each column. Values in bold indicate physician-correctly estimated risk. Values in italics indicate physician-underestimated risk. Values in bold-italics indicate physician-overestimated risk

^‡^In 2 patients, an IPSS risk categorization could not be determined because of missing data pertaining to peripheral blast percentage

### Comparison of physician-assigned versus data-derived risk categorization

Based on data-derived IPSS categorization, 147/343 patients (42.9%) were inaccurately risk-stratified by their physician; most of these inaccurate assessments (125/147 [85.0%]) were underestimations of risk (Table 4).

In a separate analysis, in which a data-derived risk category was determined with the same system used by the physician (i.e., IPSS, DIPSS, DIPSS-Plus, or IPSS when the physician used clinical/qualitative judgment), 141/343 patients (41.1%) were inaccurately risk-stratified by their physician. According to the data-derived risk categorization, the physician-assigned risk category was an underestimation in 110/141 patients (78.0%). The majority (70.9% [78/110]) of patients whose risk was underestimated were high-risk patients incorrectly categorized as intermediate risk by their providers. The use of a scoring system resulted in accurate risk categorization in 68.9% of patients, whereas 53.5% of patients were correctly risk-stratified when the physician relied on clinical/qualitative judgment (*P* < 0.01).

### Treatment initiation at diagnosis

**Fig. 1:**
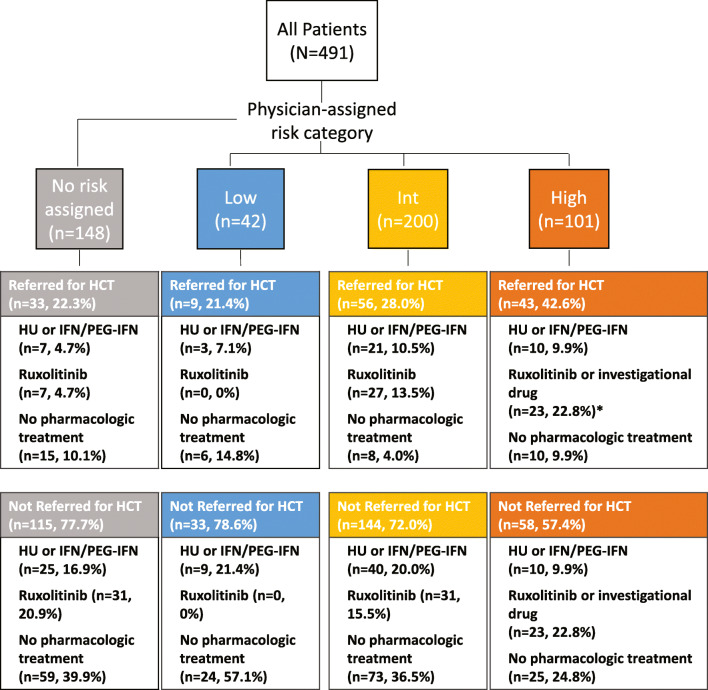
Patient flow chart. The chart shows the selection of patients into groups defined by physician-assigned risk category and patterns of treatment within 120 days of the date of MF diagnosis. Treatment categories are not mutually exclusive. *HCT* hematopoietic cell transplantation, *HU* hydroxyurea, *IFN* interferon, *Int* intermediate risk, *MF* myelofibrosis, *PEG* pegylated. Asterisk indicates that 1 patient assigned high risk received investigational treatment in a clinical trial

Of the 42 patients with physician-assigned low risk, 9 (21.4%) were referred for HCT, 12 (28.6%) were treated with HU or IFN/pegylated-IFN, and the other 30 (71.4%) did not receive any pharmacologic treatments within 120 days of diagnosis (Fig. 1). Of the 200 patients assigned intermediate risk, 56 (28.0%) were referred for HCT, 61 (30.5%) received HU or IFN/pegylated-IFN, 58 (29.0%) received ruxolitinib, and 81 (40.5%) received no pharmacologic treatment within 120 days of diagnosis. Of the 101 patients assigned high risk, 43 (42.6%) were referred for HCT, 20 (19.8%) received HU or IFN/pegylated-IFN, 45 (44.6%) received ruxolitinib, 1 (1.0%) received investigational treatments in a clinical trial, and 35 (34.7%) received no pharmacologic treatment within 120 days of diagnosis. Among the 148 patients who were not assigned a risk category by their physician, 33 (22.3%) were referred for HCT, 32 (21.6%) received HU or IFN/pegylated-IFN, 38 (25.7%) received ruxolitinib, and 74 (50.0%) received no pharmacologic treatment within 120 days of diagnosis. A total of 43/141 patients (30.5%) referred to HCT did not receive pharmacologic treatment within 120 days (Table 5). Overall, 61 patients received HCT within 120 days of diagnosis, including 1 patient assigned low risk, 10 patients assigned intermediate risk, 30 patients assigned high risk, and 20 patients who did not have a physician-assigned risk; the remaining 80 patients referred for HCT had not undergone transplant within the study timeframe.

**Table 5:** Treatment initiation at diagnosis (initiated within 120 days of the date of myelofibrosis (MF) diagnosis) by provider-assigned risk category

		Provider-assigned risk category				
	All patients (*n* = 491)	No risk score recorded (*n* = 148)	Low (*n* = 42)	Intermediate (*n* = 200)	High (*n* = 101)	Intermediate/high (*n* = 301)
Referred for HCT, *n* (%)	141 (28.7)	33 (22.3)	9 (21.4)	56 (28.0)	43 (42.6)	99 (32.9)
Any pharmacologic treatment	98 (69.5)	14 (42.4)	3 (33.3)	48 (85.7)	33 (76.7)	81 (81.8)
HU or IFN/PEG-IFN as first treatment	41 (41.8)	7 (50.0)	3 (100.0)	21 (43.8)	10 (30.3)	31 (38.3)
Ruxolitinib or investigational treatments as first treatment	57 (58.2)	7 (50.0)	0	27 (56.3)	23 (69.7)	50 (61.7)
Received HCT	61 (12.4)	20 (13.5)	1 (2.4)	10 (5.0)	30 (29.7)	40 (13.3)
Not treated	43 (30.5)	15 (45.5)	6 (66.7)	8 (14.3)	10 (23.3)	18 (18.2)
Not referred for HCT, *n* (%)	350 (71.3)	115 (77.7)	33 (78.6)	144 (72.0)	58 (57.4)	202 (67.1)
Any pharmacologic treatment	169 (48.3)	56 (48.7)	9 (27.3)	71 (49.3)	33 (56.9)	104 (51.5)
HU or IFN/PEG-IFN as first treatment	84 (49.7)	25 (44.6)	9 (100.0)	40 (56.3)	10 (30.3)	50 (48.1)
Ruxolitinib or investigational treatments as first treatment	85 (50.3)	31 (55.4)	0	31 (43.7)	23 (69.7)	54 (51.9)
Not treated	181 (51.7)	59 (51.3)	24 (72.7)	73 (50.7)	25 (43.1)	98 (48.5)

*HCT* hematopoietic cell transplantation, *HU* hydroxyurea, *IFN* interferon, *PEG* pegylated

Among patients with data-derived intermediate risk, those whose physician-assigned risk score was incorrect (including overestimations and underestimations) or underestimated only (excluding overestimations) were significantly less likely to receive any treatment (pharmacologic or HCT referral) within 120 days of diagnosis compared with patients who were correctly risk-stratified by their provider (51.6% correct vs 28.2% incorrect [*P* = 0.0134] and 18.5% underestimated [*P* = 0.0023]; Table 6 and Supplemental Table 1). The results were similar when excluding patients who received HCT (50.6% correct vs 25.7% incorrect [*P* = 0.0120] and 15.4% underestimated [*P* = 0.0014]). For patients with data-derived high risk, the rate at which treatment was received within 120 days was not significantly affected by the accuracy of the physician-assessed risk categorization (63.8% correct vs 59.1% incorrect [*P* = 0.5099] and 57.8% underestimated [*P* = 0.4142]). The results were again similar when excluding patients who received HCT (57.1% with correct risk score vs 59.8% incorrect [*P* = 0.7397] and 58.8% underestimated [*P* = 0.8423]).

**Table 6:** Treatment initiation at diagnosis (initiated within 120 days of the date of myelofibrosis (MF) diagnosis) among patients of intermediate/high risk by accuracy of physician-assigned risk category

	Data-derived intermediate risk						Data-derived high risk					
	Accuracy of physician-assigned score						Accuracy of physician-provided score					
	Total	Correct	Incorrect	*P* value*, correct vs incorrect	Underestimated	*P* value*, correct vs underestimated	Total	Correct	Incorrect	*P* value*, correct vs incorrect	Underestimated	*P* value*, correct vs underestimated
All patients†, *n*	134	95	39		27		187	94	93		83	
No pharmacologic treatment or HCT referral, *n* (%)	74 (55.2)	46 (48.4)	28 (71.8)		22 (81.5)		72 (38.5)	34 (36.2)	38 (40.9)		35 (42.2)	
Received pharmacologic treatment or HCT referral, *n* (%)	60 (44.8)	49 (51.6)	11 (28.2)	0.01	5 (18.5)	0.002	115 (61.5)	60 (63.8)	55 (59.1)	0.51	48 (57.8)	0.41
Referred for HCT	21 (35.0)	17 (34.7)	4 (36.4)		2 (40.0)		45 (39.1)	30 (50.0)	15 (27.3)		12 (25.0)	
Ruxolitinib or investigational treatments as first treatment	17 (28.3)	13 (26.5)	4 (36.4)		0		37 (32.2)	19 (31.7)	18 (32.7)		14 (29.2)	
HU or IFN/PEG-IFN as first treatment	22 (36.7)	19 (38.8)	3 (27.3)		3 (60.0)		33 (28.7)	11 (18.3)	22 (40.0)		22 (45.8)	

*HCT* hematopoietic cell transplantation, *HU* hydroxyurea, *IFN* interferon, *PEG* pegylated

^*^The difference in the proportion of patients treated versus not treated at diagnosis by correct versus underestimated risk category was assessed by chi-square test

^†^Excludes patients who were not assigned a risk score by their physician

## Discussion

Treatment recommendations for MF are guided by patient risk categorization [2]; however, this analysis of US patient records from community practices revealed that nearly one third of patients with MF did not receive a risk categorization at diagnosis. Furthermore, among patients who did receive a physician-assigned risk categorization at diagnosis, nearly one third were based on scoring systems not recommended for use at the time of diagnosis [2], and half were based on clinical judgment without use of a formal risk stratification system. When compared with data-derived IPSS risk categorization, physician-assigned risk categorization was least accurate when based on clinical/qualitative judgment (only 53.5% of patients were correctly risk-stratified when the physician relied on clinical/qualitative judgment compared with 68.9% when using a scoring system). Even among patients who received a physician-assigned risk categorization based on a scoring system, nearly one third were misclassified when compared with data-derived risk categorizations. The vast majority (85%) of misclassifications were underestimations.

Assignment of a risk classification is important for the management of MF because treatments are risk-stratified [2]. Although NCCN Guidelines for MPN were not available when the patients in this study were diagnosed, the current NCCN Guidelines and published literature support the use of IPSS at the time of MF diagnosis for risk stratification and survival estimation [2, 3, 6]. The DIPSS and DIPSS-Plus systems were intended for risk stratification and estimation of survival during the course of the disease and following treatment [2, 3, 6]. Observation is recommended for asymptomatic patients (low and intermediate 1 risks). In patients with symptomatic low-risk disease, recommended pharmacologic treatments include HU for hyperproliferative manifestations (from thrombocytosis or leukocytosis), and IFN or ruxolitinib. Ruxolitinib is also recommended for symptomatic patients with intermediate 1–risk disease and patients with intermediate 2 or high risk with platelet counts ≥ 50,000/μL who are not eligible for HCT. Fedratinib was recently approved for the treatment of adult patients with intermediate 2–risk or high-risk primary or secondary (post-PV or post-ET) MF [11]. Limiting fedratinib to patients with platelet counts ≥ 50,000/μL is recommended, including patients previously treated with ruxolitinib with no or loss of response [2]; however, approval came after the inclusion period for this study. Investigational drugs are a recommended treatment option in all MF risk categories (low risk, intermediate 1 risk, and intermediate 2 and high risks with platelet counts ≥ 50,000/μL). Referral for evaluation of HCT is a recommended treatment option for all patients except those with low-risk disease. Misclassification of patients may result in misaligned treatment strategies. Patients with intermediate risk whose physician-assessed risk was underestimated were significantly less likely to receive treatment within 120 days following diagnosis than patients who received the correct risk score. Although some high-risk patients were undercategorized by their provider as intermediate risk, they were likely to still receive recommended treatments because treatment recommendations for high- and intermediate-risk MF are similar [2]. Of the patients classified as low risk, 71.4% received no pharmacologic treatment. Although no treatment may be appropriate for asymptomatic patients with low-risk MF, 76.2% (32/42) of the patients who were classified as low risk by a physician were determined (based on data) to have either intermediate or high risk. Furthermore, while HU and IFN are not recommended for patients with intermediate 2 risk, patients in this risk category received HU or IFN at similar rates as ruxolitinib or investigational drugs, despite a demonstrated survival advantage with ruxolitinib in the phase 3 COMFORT-1/2 clinical trials [12, 13]. Of patients assessed as low risk by a treating physician, 21.4% were referred for HCT, a treatment option that is not recommended for patients with low risk. It should be noted that there might be situations in which deviating from NCCN Guidelines–recommended therapy is appropriate. For example, many MF treatments can lower blood counts, and low counts (especially anemia) are a major issue in MF [6, 14]. Therefore, physicians may choose not to start therapy recommended by NCCN owing to low or borderline blood counts [14]. Likewise, various considerations for HCT, such as patient age, race, insurance coverage, and donor matching, can result in low levels of referral when compared with guideline recommendations [15, 16].

The limitations of this study are consistent with a retrospective medical chart review, such as potential for patient selection bias at sites and record completeness. The sample size for patients whose risk was overestimated was too small to allow for a rigorous statistical analysis. Most patients in the study were diagnosed with primary MF, for which the use of IPSS, DIPSS, and DIPSS-Plus has been validated [2]. These risk assessment tools have not been validated in post-ET and post-PV MF [2], and other tools have been proposed for use with these patients [9].

## Conclusions

Analysis of chart records revealed potential improvements for the management of MF, especially concerning risk stratification at diagnosis. Risk stratification is recommended for patients with MF to guide treatment choice, yet no risk categorization was assigned at diagnosis for approximately 30% of patients included in this study. When medical records were used to assign data-derived risk classifications and compared with the physician-assigned risk categorizations at the time of MF diagnosis, approximately 40% of the physician-assigned categorizations were found to be inaccurate. However, assignment of an accurate risk categorization did not guarantee that patients received recommended treatment because most patients with intermediate- or high-risk disease did not initiate treatment within 120 days of diagnosis. Of those patients who received pharmacologic treatment, half were treated with HU or IFN, which are not recommended for the treatment of intermediate- and high-risk patients. Among patients with intermediate-risk disease, those whose risk was underestimated by their provider were less likely to initiate recommended pharmacologic treatment compared with those who received a correct risk categorization. Treatment recommendations are risk-stratified, and therefore appropriate treatment strategy is dependent upon accurate risk categorization. Physician education on NCCN Guidelines and recommendations may improve overall patient management and outcomes.

## Electronic supplementary material

ESM 1 (DOCX 21 kb)
