# Supplementary material for: Real-world risk assessment and treatment initiation among patients with myelofibrosis at community oncology practices in the United States
Source: Ann Hematol. 2020 May 7;99(11):2555–64. doi: 10.1007/s00277-020-04055-w (PMC7536164; doi:10.1007/s00277-020-04055-w)
Supplement: Supplementary file 1 — (DOCX 21 kb) [file 277_2020_4055_MOESM1_ESM.docx]

# **Supplemental Materials**

**Table 1. Treatment Initiation at Diagnosis* Among Patients With Intermediate and High Risk by Accuracy of Physician-Assigned Risk Category**

|  | **Data-Derived Intermediate Risk** | | | | | | **Data-Derived High Risk** | | | | | |
| --- | --- | --- | --- | --- | --- | --- | --- | --- | --- | --- | --- | --- |
|  |  | **Accuracy of Physician-Assigned Score** | | | | |  | **Accuracy of Physician-Provided Score** | | | | |
|  | **Total** | **Correct** | **Incorrect** | ***P* Value, ^†^ Correct vs Incorrect** | **Underestimated** | ***P* Value,^†^ Correct vs Underestimated** | **Total** | **Correct** | **Incorrect** | ***P* Value,^†^ Correct vs Incorrect** | **Underestimated** | ***P* Value,^†^ Correct vs Underestimated** |
| All patients,**^‡^** n | 134 | 95 | 39 |  | 27 |  | 187 | 94 | 93 |  | 83 |  |
| No pharmacologic treatment or HCT referral, n (%) | 74 (55.2) | 46 (48.4) | 28 (71.8) |  | 22 (81.5) |  | 72 (38.5) | 34 (36.2) | 38 (40.9) |  | 35 (42.2) |  |
| Received pharmacologic treatment or HCT referral, n (%) | 60 (44.8) | 49 (51.6) | 11 (28.2) | 0.01 | 5 (18.5) | 0.002 | 115 (61.5) | 60 (63.8) | 55 (59.1) | 0.51 | 48 (57.8) | 0.41 |
| Referred for HCT | 21 (35.0) | 17 (34.7) | 4 (36.4) |  | 2 (40.0) |  | 45 (39.1) | 30 (50.0) | 15 (27.3) |  | 12 (25.0) |  |
| HU or IFN/PEG-IFN as first treatment | 22 (36.7) | 19 (38.8) | 3 (27.3) |  | 3 (60.0) |  | 33 (28.7) | 11 (18.3) | 22 (40.0) |  | 22 (45.8) |  |
| Ruxolitinib or investigational treatments as first treatment | 17 (28.3) | 13 (26.5) | 4 (36.4) |  | 0 |  | 37 (32.2) | 19 (31.7) | 18 (32.7) |  | 14 (29.2) |  |
| All patients, excluding those who received HCT,^§^ n | 124 | 89 | 35 |  | 26 |  | 157 | 70 | 87 |  | 80 |  |
| No pharmacologic treatment or HCT referral, n (%) | 70 (56.5) | 44 (49.4) | 26 (74.3) |  | 22 (84.6) |  | 65 (41.1) | 30 (42.9) | 35 (40.2) |  | 33 (41.3) |  |
| Received pharmacologic treatment or HCT referral, n (%) | 54 (43.5) | 45 (50.6) | 9 (25.7) | 0.012 | 4 (15.4) | 0.001 | 92 (58.6) | 40 (57.1) | 52 (59.8) | 0.74 | 47 (58.8) | 0.84 |
| Referred for HCT | 16 (29.6) | 14 (31.1) | 2 (22.2) |  | 1 (25.0) |  | 22 (23.9) | 10 (25.0) | 12 (23.1) |  | 11 (23.4) |  |
| HU or IFN/PEG-IFN as first treatment | 21 (38.9) | 18 (40.0) | 3 (33.3) |  | 3 (75.0) |  | 33 (35.9) | 11 (27.5) | 22 (42.3) |  | 22 (46.8) |  |
| Ruxolitinib or investigational treatments as first treatment | 17 (31.5) | 13 (28.9) | 4 (44.4) |  | 0 |  | 37 (40.2) | 19 (47.5) | 18 (34.6) |  | 14 (29.8) |  |
| Patients referred for HCT, n | 37 | 27 | 10 |  | 3 |  | 60 | 37 | 23 |  | 19 |  |
| Received HCT within 120 days, n (%) | 10 (27.0) | 6 (22.2) | 4 (40.0) |  | 1 (33.3) |  | 30 (50.0) | 24 (64.9) | 6 (26.1) |  | 3 (15.8) |  |
| Treated with ruxolitinib or clinical trial | 2 (20.0) | 1 (16.7) | 1 (25.0) |  | 0 |  | 13 (43.3) | 12 (50.0) | 1 (16.7) |  | 0 |  |
| Referred but did not receive HCT, n (%) | 27 (73.0) | 21 (77.8) | 6 (60.0) |  | 2 (66.7) |  | 30 (50.0) | 13 (35.1) | 17 (73.9) |  | 16 (84.2) |  |
| Treated with ruxolitinib or investigational treatments | 8 (29.6) | 7 (33.3) | 1 (16.7) |  | 0 |  | 17 (56.7) | 9 (69.2) | 8 (47.1) |  | 7 (43.8) |  |

HCT, hematopoietic cell transplantation; HU, hydroxyurea; IFN, interferon; MF, myelofibrosis; PEG, pegylated.
* Initiated within 120 days of the date of MF diagnosis. ^†^ The difference in the proportion of patients treated versus not treated at diagnosis by correct versus underestimated risk category was assessed by chi-square test.

^‡^ Excludes patients who were not assigned a risk score by their physician. ^§^ 40 patients excluded, intermediate risk, n=10; high, n=30.
